# High-addition segmented refractive bifocal intraocular lens in inactive age-related macular degeneration: A multicenter pilot study

**DOI:** 10.1371/journal.pone.0256985

**Published:** 2021-09-02

**Authors:** Gerd U. Auffarth, Josef Reiter, Martin Leitritz, Karl-Ulrich Bartz-Schmidt, Fabian Höhn, Detlev Breyer, Hakan Kaymak, Klaus Rohrschneider, Ramin Khoramnia, Timur M. Yildirim

**Affiliations:** 1 International Vision Correction Research Centre, Department of Ophthalmology, University of Heidelberg, Heidelberg, Germany; 2 Augen-MVZ Landshut, Landshut, Germany; 3 Centre for Ophthalmology, University of Tübingen, Tübingen, Germany; 4 Marienhospital Osnabrück, Osnabrück, Germany; 5 Breyer & Kaymak Augenchirurgie, Düsseldorf, Germany; University of Toronto, CANADA

## Abstract

This multicenter, open-label study aimed to determine the safety and functional outcome of a high-addition segmented refractive bifocal intraocular lens (IOL) in late inactive age-related macular degeneration (AMD). Twenty eyes of 20 patients were enrolled and followed until 12 months after the intervention. Patients underwent cataract surgery with implantation of a LS-313 MF80 segmented refractive bifocal intraocular lens with a near addition of +8.0 D (Teleon Surgical Vertriebs GmbH, Berlin, Germany). The main outcome measures were distance corrected near visual acuity (DCNVA) and safety as determined by intra- and post-operative complications. Secondary outcomes included distance corrected visual acuity (CDVA), uncorrected distance visual acuity (UDVA), uncorrected near visual acuity (UNVA), the need for magnification to read newspaper, preferred reading distance, speed and performance (logRAD), as well as patient satisfaction. Mean DCNVA improved from 0.95 (±0.19) to 0.74 (±0.35) logMAR, until 6 months after surgery, P<0.05. CDVA improved from 0.70 (±0.23) to 0.59 (±0.30) logMAR, UDVA from 0.94 (±0.25) to 0.69 (±0.34) logMAR, UNVA from 1.08 (±0.19) to 0.87 (±0.43) logMAR. The mean need for magnification decreased from 2.9- to 2.3-fold, preferred reading distance from 23 to 20 cm. No intraoperative complications occurred during any of the surgeries. One patient lost > 2 lines of CDVA between 6 and 12 months, in another case, the study IOL was exchanged for a monofocal one due to dysphotopsia and decreased CDVA. Implantation of a segmented refractive bifocal IOL with +8.0 D addition improves near and distance vision in patients with late AMD and has a satisfactory safety profile.

## Introduction

Age-related macular degeneration (AMD) is the main cause of blindness in high-income countries [1]. The Age-Related Eye Disease Study (AREDS) classifies AMD depending on the location, size, area and type of drusen, pigmentation and geographic atrophy in a standard macular grid with circles [2]. The National Institute for Health and Care Excellence (NICE) recommends classifying the disease in one early and four late types (indeterminate, wet active, dry, wet inactive) [3]. Intravitreal therapy with vascular endothelial growth factor (VEGF) antibodies has profoundly improved the treatment of wet active AMD, but this type only affects about 10% of patients [4]. For the remaining AMD population, there is currently no effective treatment and thus there is an unmet need for options that improve their quality of vision [5]. Today, the main approach to help patients cope with their reduced vision and enable them to handle everyday tasks is visual rehabilitation with external low-vision aids like spectacles, hand-held telescopes or magnifiers. However, such aids have several drawbacks, reducing the visual field, restricting movement and the necessity of extensive training in the use of the aids [6].

There have been specialized intraocular lens (IOL) implants developed to improve AMD patients’ vision and act as alternatives to external aids. These IOLs either magnify or eccentrically dislocate the image projected on the retina or attempt to combine both magnification and image-displacement [6–14]. Earlier devices were rigid and bulky and implanted through a large incision of 5 to 12 mm. More modern IOLs are made from foldable acrylic allowing implantation through much smaller incisions of 2.2 to 3 mm [6].

One modern device with an optical concept new to the field of AMD treatment, is a high-addition (+8 D) segmented refractive bifocal IOL. Optical bench studies confirm good image quality in vitro [15]. IOLs using the same optical principle with lower additions, are already frequently used in cataract and refractive lens exchange. The clinical functional performance and satisfaction of patients asking for spectacle independence are good [16, 17]. However, as the high-addition model of this IOL is intended to be used to treat AMD patients—a very different patient group to cataract and refractive patients—it is important to get clinical data. Until recently, only a single case report has provided the first clinical data about this implant—reporting major benefits in a patient with end-stage AMD [18]. Even though this conclusion is promising, there is a lack of clinical data that provides information about safety and efficacy of this new IOL implant.

We aimed to determine the safety and functional outcome after implantation of this high-addition segmented refractive bifocal IOL for treating patients with late inactive AMD.

## Methods

### Patient enrollment

We enrolled patients in this multicenter, open-label clinical study, between January 2017 and August 2018. All patients had late inactive age-related macular degeneration (AMD), which was classified according to the NICE guideline [3]. As this was a pilot study on a new treatment concept, patients had to meet conservative study criteria: the study eye had to be diagnosed with AMD and require an IOL power between +15.0 to +25.0 D to target emmetropia [3]. Preoperative and expected postoperative corrected distance visual acuity (CDVA) had to range from 1.3–0.4 logMAR in both eyes. We excluded cases with previous corneal surgery or scars, preoperative corneal astigmatism over 2.0 D, uveitis, congenital retinal dystrophies, or only one eye, and patients with conditions complicating an in-the-bag IOL implantation (e.g. pseudoexfoliation syndrome or zonulolysis).

All patients gave written informed consent to participation in this study, which it was conducted in accordance with internationally recognized guidelines, including Good Clinical Practice (ICH-GCP) and the Declaration of Helsinki. The Ethics Committee of the University of Heidelberg approved this study (S-213/2016).

### Intraocular lens implant

**Fig 1:**
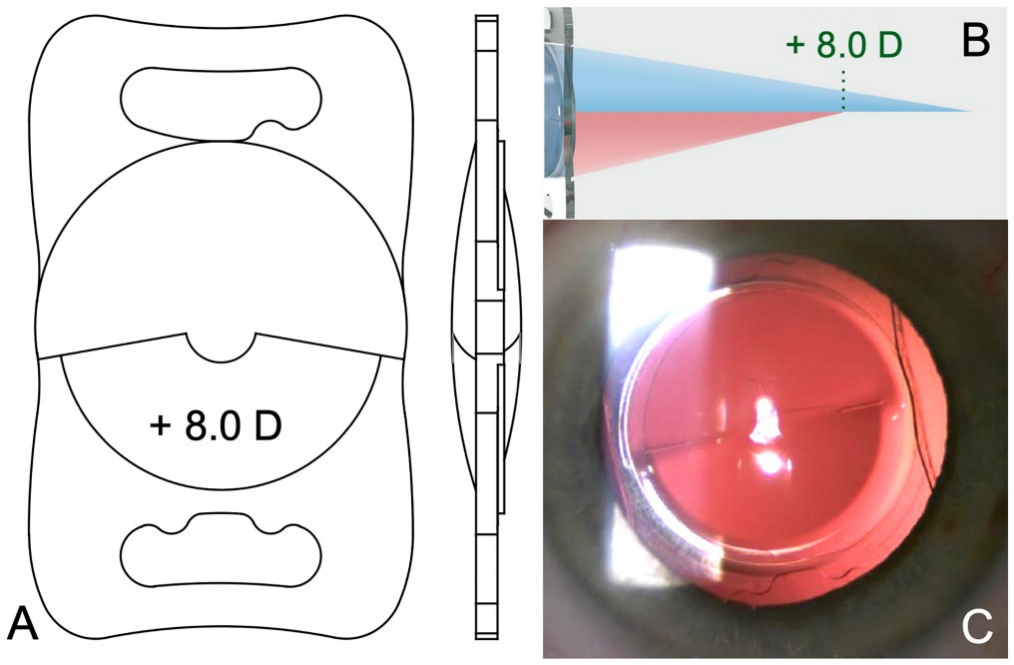
The high-addition segmented refractive bifocal study intraocular lens (IOL). Schematic images of the plate-haptic IOL with high-addition refractive segments embedded in the inferior anterior and posterior parts of the lens (**A**). The refractive optical principle allocates incoming light to a near and a distant focus (**B**). Reprinted with permission from Teleon Surgical Vertriebs GmbH, Berlin, Germany. A slit-lamp photograph shows the Study IOL in a patients’ eye (**C**).

The eye with the better visual prognosis underwent cataract surgery with implantation of the study IOL, the Lentis LS-313 MF80 (Teleon Surgical Vertriebs GmbH, Berlin, Germany), a segmented refractive bifocal intraocular lens with a near addition of + 8.0 D, designed to offer approximately 2.5-fold magnification at 30 cm reading distance. The LS-313 MF80 (henceforth called ‘study IOL’), is rotationally asymmetrical IOL with an aberration-neutral segmented refractive bifocal optic that has 360° sharp-edge plate haptics (Fig 1).

It is implanted in the capsular bag like any other plate-haptic-IOL during routine cataract surgery through a 2.2 mm clear corneal or limbal incision (S1 Video).

### Follow-up and outcome measures

Follow up examinations were within 1 week, and 2, 6 and 12 months after surgery. At each study visit, eyes were examined using slit-lamp microscopy: at the enrolment visit and at the 6- and 12-month examinations, and in case of any suspected macular changes, optical coherence tomography follow-up images were obtained to monitor the AMD. The main outcome measure was distance corrected near visual acuity (DCNVA). Secondary outcome included CDVA, UDVA, UNVA, need for magnification to read newspaper, preferred reading distance, reading speed and reading performance (logRAD and logRAD score).

We assessed reading performance using two reading charts that consist of texts in different font size.: Schweizer (Schweizer GmbH, Forchheim, Germany) and Radner (Oculus, Wetzlar, Germany) [19]. We used Schweizer charts to assess the Patient’s need for magnification for the study eye. The Patient read the charts at 25 cm distance with their best distance correction and a +4.0 D addition. The required near addition was determined by multiplying the need for magnification by four. Using the Radner charts, the reading performance (reading acuity, preferred reading distance and speed) was assessed with the patients’ distance correction and required near addition. The reading speed, given in words per minute, was tested at 0.2 logRAD larger than the smallest readable line to ensure comparable results for all patients.

Six months after surgery, patients were asked to complete a questionnaire (in German) with ten questions about their subjective satisfaction and reading habits.

### Safety measures

We assessed safety as determined by intra- and postoperative complications, including the course of intraocular pressure (IOP) and the number of patients with loss of more than two lines of CDVA. Furthermore, as multifocal IOLs are considered to induce dysphotopsia such as glare and halos, the subjective questionnaire included questions on the perception of double vision, glare, halos and starburst.

### Data analysis

Data were collected in an Excel 16.44 spread sheet (Microsoft Excel, Redmond, USA) and given as mean (±SD) or median (range). All data of patients who received the study IOL were included in the analysis. As DCNVA data satisfied the assumption of normality distribution (Kolmogorov-Smirnov) and equality of variance (Levene), different time points were compared using the one-way ANOVA. The remaining data was analyzed using the Friedman test. A P value of < 0.05 was considered statistically significant.

## Results

### Study population

**Fig 2:**
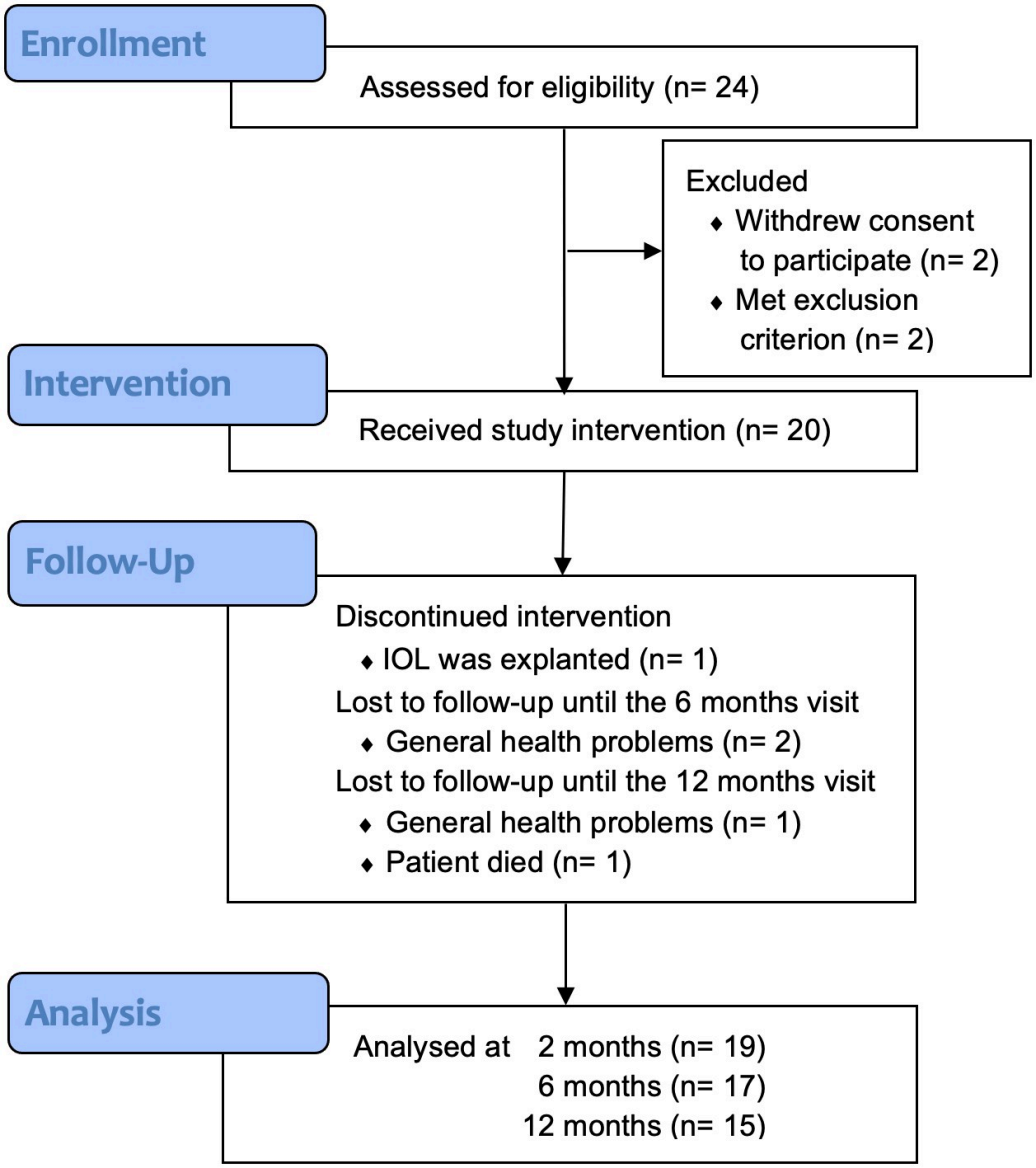
Flow chart showing the progress of the study. Twenty-four patients were included in the study. After two patients withdrew consent and two met an exclusion criterion, twenty eyes received the study IOL. The reasons for drop-out after receiving the intervention and the number of analyzed data-sets are given for each time-point.

Twenty-four patients were included to the study, twenty received the study IOL, nineteen were included for analysis at two months after surgery, seventeen at six months and this reduced to fifteen at the twelve-month examination (Fig 2).

**Fig 3:**
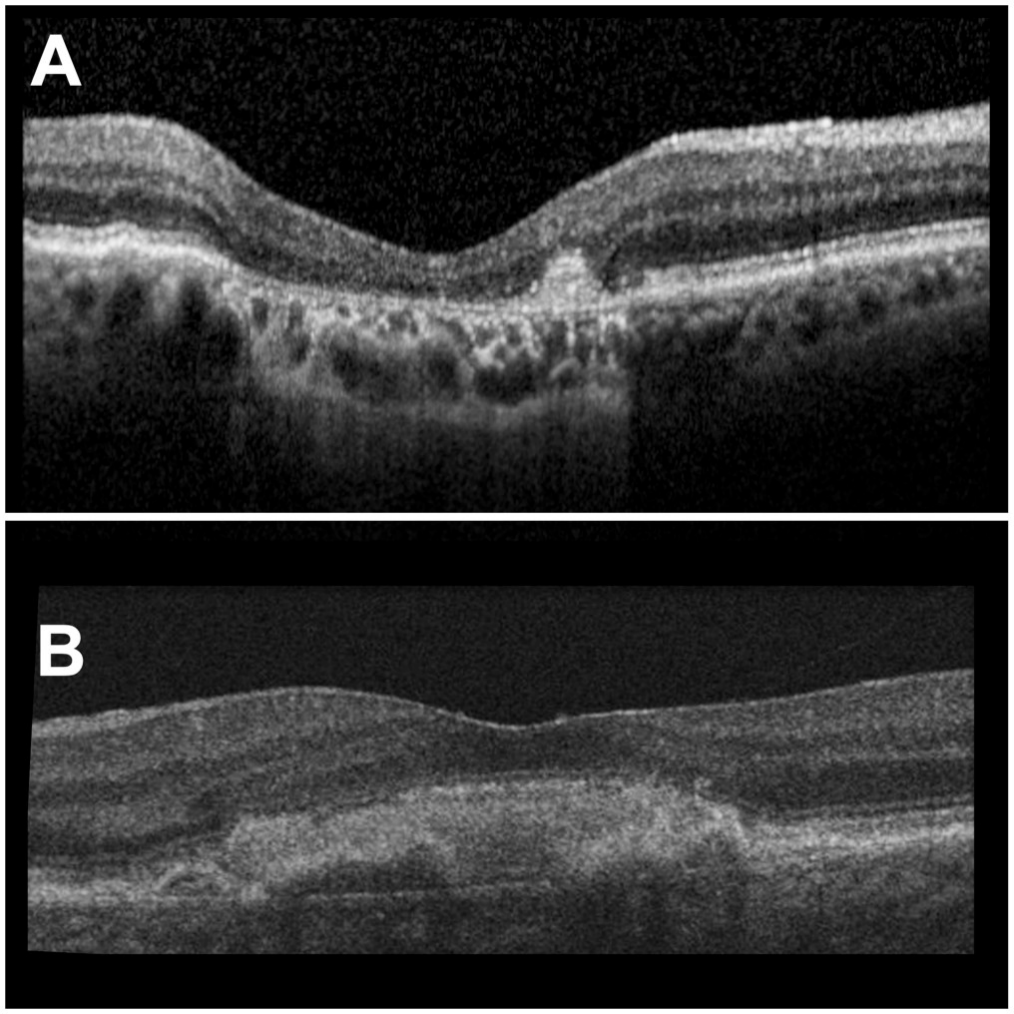
Optical coherence tomography B-scans through the fovea of 2 study eyes with different types of age-related macular (AMD) degeneration. Ten eyes (50%) were classified as late-stage dry (**A**), six eyes (30%) as late-stage wet inactive (**B**) AMD. **A**: Geographic atrophy with a destructed photoreceptor layer. **B**: Subretinal fibrosis with hyperreflectivity.

The mean age of patients receiving the study IOL was 77 (±8.7) years. Twelve right eyes and eight left eyes of twelve female and eight male patients were treated. The majority of eyes were classified as late dry and late wet inactive AMD, with 10 (50%) and 6 (30%) eyes, respectively (Fig 3).

The remaining 4 (20%) eyes were classified as late indeterminate. The preoperative spherical equivalent (SEQ), axial length and pupil size, IOL power and expected postoperative SEQ were 0.6 (±2.4) D, 23.5 (±0.6) mm, 3.7 (±1.1) mm, 21.0 (18.0–24.5) D, -0.2 (±0.22) D, respectively.

### Functional vision outcome

**Fig 4:**
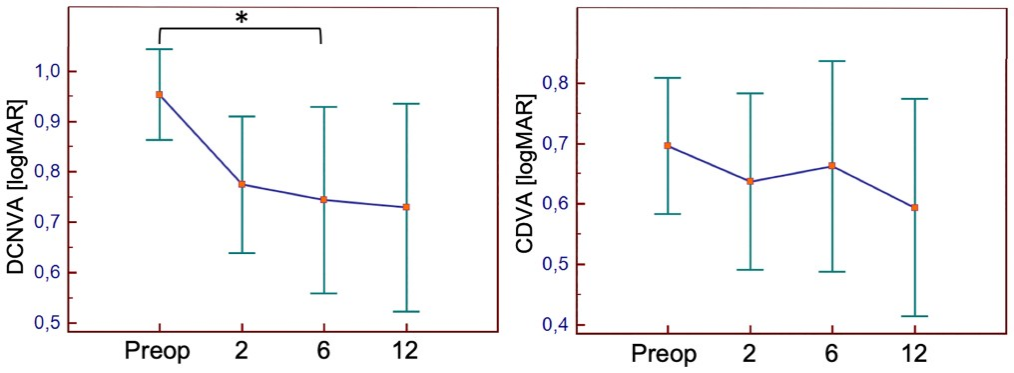
The course of distance corrected near visual acuity (DCNVA) throughout the study. *Indicates statistically significant difference, one-way ANOVA, P<0.05.

DCNVA improved statistically significant from 0.95 (±0.19) to 0.74 (±0.35) logMAR until six months after surgery and remained stable at one year postoperatively (Fig 4), P<0.05.

**Fig 5:**
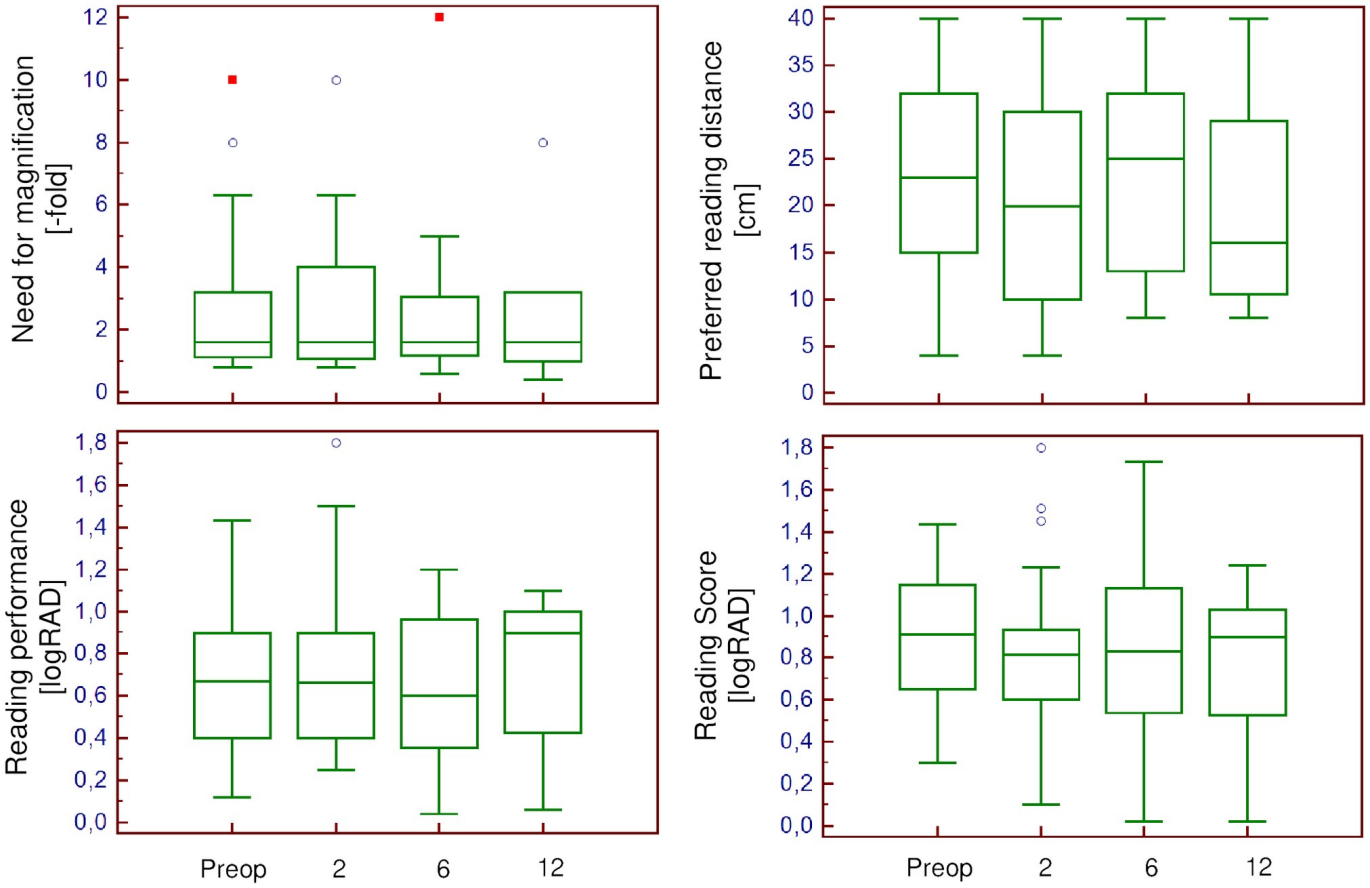
Secondary outcome parameters. The course of the need for magnification, preferred reading distance, reading performance and reading scores throughout the study.

CDVA improved from 0.70 (±0.23) to 0.59 (±0.30) logMAR, UDVA from 0.94 (±0.25) to 0.69 (±0.34) logMAR, UNVA from 1.08 (±0.19) to 0.87 (±0.43), until 12 months after surgery. The need for magnification decreased from 2.9 (±2.6)- to 2.3 (±1.8)-fold and preferred reading distance from 23 (±11) to 20 (±10) cm, until 12 months after surgery. Reading acuity and speed, as well as the logRAD score remained similar (Fig 5).

### Subjective questionnaire

Six months after surgery, patients reported that their quality of vision and distant vision was rather good, with 80% of patients claiming to have an overall good or acceptable quality of vision. Fifty-seven percent of patients reported they did not require additional aids for reading large font (Table 1).

**Table 1:** Quality of vision subjective questionnaire.

Item	Mean (±SD)	Scale
1. Overall quality of vision	44.3 (±26.8)	(0, perfect to 100, very poor)
2. Quality of distant vision	45.0 (±22.7)	(0, perfect to 100, very poor)
3. Quality of intermediate vision	50.7 (±23.5)	(0, perfect to 100, very poor)
4. Quality of near vision without aids	61.3 (±29.9)	(0, perfect to 100, very poor)
5. Reading newspaper without aids	83.3 (±24.4)	(0, perfect to 100, very poor)
6. Reading large font without aids	41.4 (±48.1)	(0, not at all to 100, very strong)
7. Perception of glare	49.3 (±35.7)	(0, none to 100, very strong)
8. Perception of halos	22.1 (±29.1)	(0, none to 100, very strong)
9. Perception of starburst	19.3 (±31.0)	(0, none to 100, very strong)
10. Double vision	1.8 (±5.2)	(0, none to 100, very strong)

### Safety

There were no intraoperative complications during any of the implantation surgeries. The postoperative course was mostly uneventful and within the expected limits of routine follow-up of cataract surgery. The mean IOP was preoperatively 15.1 (±2.4) then 15.7 (±3.0) at 1–7 days, 14.5 (±2.7) at 2 months, 14.7 (±3.9) at 6 months and 15.2 (±2.2) at 12 months after surgery. One patient lost > 2 lines of CDVA between 6 and 12 months due to progression of the macular disease. In one case, the study IOL was exchanged for a CT Spheris 209M monofocal IOL (Carl Zeiss Meditec AG, Berlin, Germany) 20 days after the initial surgery due to pronounced subjective dysphotopsia and a decrease in CDVA from 0.4 preoperatively to 0.54. One patient reported mild double vision, 28% reported some degree of starburst, 28% halos and 64% glare.

## Discussion

The Study IOL is based on a segmented refractive bifocal optical principle initially developed for the use in refractive surgery [16, 17, 20]. Such implants with additions ranging from + 1.5 to + 3 D, are widely used in Europe and Asia to provide patients with spectacle independence [16, 17]. In an early clinical study, Kretz et al. investigated the performance of the Lentis Comfort (+ 1.5 D addition), in sixty eyes of thirty patients [17]. The mean postoperative values for CDVA and DCIVA were -0.08 and -0.02 logMAR, respectively. The authors concluded that the Lentis Comfort offers good distance visual function in combination with a good intermediate functional performance [17]. In another prospective clinical study, Linz et al. investigated the Lentis Mplus (toric), having + 3.0 D addition [16]. The median CDVA and CNVA were 0.01 and 0.03 logMAR, respectively [16]. With implementing a much higher near segment addition compared to the refractive IOL models, the current Study IOL was designed to be implanted in AMD eyes as an alternative to external reading aids that are rather difficult to handle for a patient. To measure the Study lens’ optical performance we formerly conducted an in vitro study at the David J. Apple International Laboratory for Ocular Pathology [15]. Using an optical bench setup, the LS-313 MF80 was compared to the predecessor models, the Lentis Comfort and the Lentis Mplus, that are used in refractive surgery for patients requesting spectacle independence [15]. With a 3.0 and 4.5 mm aperture, the MF80 outperformed both lenses at the near and the distant focus; but as expected, at the intermediate focus, the refractive implants yielded better results [15]. This laboratory study, encouraged us to further investigate this therapeutic principle for AMD patients.

First clinical results of this implant have been presented as a case report in 2019 by Borkenstein and Borkenstein, who described a 86-year-old patient with late-stage AMD in both eyes [18]. Cataract surgery with implantation of the LS-313 MF80 performed in one eye with simultaneous intravitreal injection of an anti-vascular endothelial growth factor agent improved CDVA and DCNVA [18]. In a subsequent study by the same authors, 4-years observational outcomes of 11 patients treated with this implant were reported [21]. The authors found an increase in CDVA and UNVA in all cases and 10/11 patients reported an improved quality of life. The authors claimed that this IOL offers safe and durable functional and quality of life benefits. They stated that additional clinical research would be needed to collect more outcome data to determine the clinical value of this implant [21].

In our presented clinical study on 20 eyes of 20 patients, mean DCNVA improved from 0.95 (±0.19) to 0.74 (±0.35) logMAR, until 6 months after surgery, P<0.05. Important safety parameters were satisfactory, including the rate of explantation, loss of CDVA and rise of IOP. However, some patients reported the perception of photopic phenomena, especially glare, which is an uncommon complaint for refractive segmented IOL models and has not been noted in previous studies on similar IOL models [16, 17]. One might speculate that the dysphotopsia arose from the macular disease itself but we would have to assess this in future studies of this implant.

The improvement of near vision is especially relevant to elderly patients: as previous studies have demonstrated, near vision impairment, but not distance vision or hearing impairment were associated with cognitive decline in older patients [22]. Near vision leisure activities like reading, playing board games or playing musical instruments have been shown to be associated with a lower risk of developing dementia by increasing the cognitive reserve and delaying the clinical or pathological onset [23]. Furthermore, as the elderly are often restricted in their movement due to musculoskeletal comorbidities, a greater proportion of time is spent on near vision rather then distance vision activities like biking or hiking, that require a healthier musculoskeletal state [24]. Therefore, improving old patients’ near vision is of particular interest to maintaining their general health and wellbeing. However, external magnifying lenses and other reading aids are sometimes difficult to handle. In order to achieve freedom from external aids, IOL implants have been developed based on different optical principles to improve functional vision in AMD patients [6–13]. Some of such IOLs, like the IMT or the IOL-VIP, are quite large multipiece devices associated with a high rate of complications [7, 11]. The IMT and IOL-VIP are based on the optical principle of an intraocular Galilean telescope with two lenses, one converging- and one diverging-lens, that act together to create image magnification. A study investigating the IMT found that 14 of 40 eyes experienced adverse effects of varying degree, including five cases of explantation due to patient dissatisfaction or bubbles developing inside the IMT, two cases with transient hypopyon, two cases with damage to the iris, one case of zonule rupture and one case of iris sphincter erosion [7]. Compared to the IMT, the LS-313 MF80 seems to provide superior safety, without intraoperative or postoperative mechanical complication. Only one case required explantation due to patient dissatisfaction. Alio et al. presented a study on 40 eyes of 40 patients with dry AMD treated with an IMT [7]. Patients were followed for 12 months after surgery, in which mean preoperative uncorrected distance VA (UDVA) in the operated eye improved from 0.9 to 0.6 logMAR [7]. In our study, the UDVA improvement was similar, from 0.94 to 0.69 logMAR. The greater amount of complications with the IMT can be explained by its larger size compared to an IOL, which when implanted leads more mechanical irritation with adjacent tissues [6, 7]. In a large prospective multicenter clinical trial, Boyer et al. investigated the long-term results of 217 patients treated with the IMT for bilateral end-stage AMD [25]. The mean CDVA improvement from baseline to 60 months was 2.4 lines. Quality of life scores were significantly higher in the younger patient group (65 to >75 years). In this study, several complications have been reported, including iritis, persistent corneal edema, corneal transplantation, IMT removal and loss of > 2 lines of CDVA [25]. However, it should be noted that the IMT is presently available in a less invasive version. Another Galilean telescope-type implant is the IOL-VIP [10, 11, 26]. Orzalesi et al. presented a case series with forty consecutive surgical and rehabilitative procedures. The mean CDVA improved from 1.28 to 0.77 logMAR, without any intraoperative or postoperative complications [26]. A more recent study by Dag et al. from 2019 reported results of thirteen eyes. Pre- and postoperative CDVA were 1.08 and 0.81 logMAR in the operated eye, respectively. However, while VA and quality of life increased, the mean endothelial cell density decreased from 2437 preoperatively to 1849 at three month after surgery [10]. The iolAMD (London Eye Hospital Pharma, London, UK), another Galilean-type-implant is made from two foldable hydrophobic acrylic IOLs implanted through a much smaller incision. First results on three eyes treated with the iolAMD by Hengerer et al. in 2015 were promising [12]. A prospective intervention pilot study was presented by Quereshi et al., that included twelve patients that were followed for 4 months. The mean decimal CDVA improved from 0.12 to 0.20 four months after surgery, without significant intraoperative or postoperative complications [27]. As a successor of the iolAMD, the EyeMax mono (London Eye Hospital Pharma, London, UK) approved in 2016 is based on a hyperaspheric, single-piece, hydrophobic acrylic IOL platform, designed to increase the quality of the image on the macula at ≤10° of retinal eccentricity and generate a magnification of approximately 1.1- to 1.2- fold [6, 13]. Hengerer et al. treated twenty-two pseudophakic eyes with AMD by implanting the EyeMax mono into the ciliary sulcus [13]. No intraoperative complication occurred and all eyes gained > 2 lines of CDVA by 6 months after surgery. The authors concluded that the EyeMax mono IOL is safe and has the potential to improve near and distance visual acuity in pseudophakic eyes with intermediate to severe AMD [13]. Apart from Galilean telescope-type devices, there are implants incorporating other optical principles. The Lipshitz Mirror implant, LMI (Optolight Vision Technology, Herzlia, Israel) uses two miniature mirrors to create a Cassegrain telescope, providing a 2.5-fold magnification on the central retinal [9, 28]. The Fresnel Prism IOL (Rayner Intraocular Lenses Limited, Hove, UK) incorporates a Fresnel prism at the posterior surface of the IOL optic that leads to a 6° deviation of light that enters the eye and displaces the retinal image [14]. Clinical studies on such implants are rather scarce. A pseudophakic approach is the supplementary one-piece hydrophobic acrylic Scharioth macula lens, SML (Medicontur, Zsámbék, Hungary) [8]. In 2019, Srinivasan reported a study on fifty pseudophakic eyes with inactive AMD, treated with the SML, with no intraoperative complications [29]. Similar to our study, one IOL required explantation due to postoperative perception of halos and glare. While the mean decimal CNVA improved from 0.23 (± 0.12) to 0.57 (±0.33) one year after surgery, the mean CDVA remained unchanged at 0.19 decimal [29].

Regarding functional outcomes, our results shows a trend towards better reading performance until the six months visit. An interesting finding from our study was the decrease of the preferred reading distance. In theory, with a +8.0 D addition, the optimal distance for a reading would be 12.5 cm. However, our results show that the average preferred reading distance only decreased from 23 (±11) to 20 (±10) cm, until 12 months after surgery. This finding suggests that the subjects chose a compromise between their habitual reading distance and the distance that is optimal for their +8.0 D implant. One can therefore expect that patients might profit from additional rehabilitation training to learn how to optimize their habits with the new optics after the surgery. As the main aim of this pilot study was to evaluate safety and VA of this new treatment concept, the number of patients was calculated on the basis of the primary endpoint, DCNVA. However, a larger study might also reveal statistically significant benefits regarding reading performance or other functional outcome measures. Previous studies on multifocal IOLs suggested that these work best if they are implanted in both eyes [30]. Because the concept of a refractive bifocal IOL for AMD treatment was new, in this pilot study we restricted implantation to one eye only. However, binocular implantation might be a reasonable option to improve the functional outcome, especially as the device does not restrict the field of vision. Future studies should investigate, whether binocular implantation could offer additional advantages in regard to relevant functional outcome measures, like reading performance, apart from the improvement in DCNVA. However, one has to take into account that the high magnification could result in problems with binocular vision due to the strong convergence required. The presented study has some limitations: it lacks a randomized control group of patients treated with monofocal IOLs. The sample size was rather small, so larger studies are needed to confirm our results. As the follow-up period was limited to one year, our study does not provide long-term data on this implant, which should be assessed in future studies. One concern for clinicians could also be binocular function, which was not specifically assessed in this study. Furthermore, we used a non-validated subjective questionnaire to assess patient-reported outcome measures.

Nevertheless this study provides important clinical data on a segmented refractive bifocal IOL implant with a +8.0 D addition to treat patients with late-stage AMD. This therapeutic option seems to have a superior safety profile compared to other devices from this field and offer good functional outcome results. Monocular implantation of the LS-313 MF80 improved near and distance vision, providing an option for the visual rehabilitation of patients with late-stage AMD. This novel IOL offers an alternative to improve functional vision in patients that are usually dependent on complicated external vision aids.

## Supporting information

S1 Video The implantation of the study IOL. The loading in the injector and lens implantation is comparable with that of a standard hydrophilic acrylic plate-haptic single-piece IOL.
(MP4)
